# Estimating the burden of malaria in pregnancy: a case study from rural Madhya Pradesh, India

**DOI:** 10.1186/1475-2875-8-24

**Published:** 2009-02-12

**Authors:** Nadia Diamond-Smith, Neeru Singh, RK Das Gupta, Aditya Dash, Krongthong Thimasarn, Oona MR Campbell, Daniel Chandramohan

**Affiliations:** 1 London School of Hygiene and Tropical Medicine, London, UK; 2 Regional Malaria Research Centre for Tribals, Jabalpur, India; 3 National Vector Borne Disease Control Programme, Delhi, India; 4 National Institute for Malaria Reseach, Delhi, India; 5 World Health Organization, South East Asia Regional Office, Delhi, India

## Abstract

**Background:**

Malaria in pregnancy (MiP) is inadequately researched in India, and the burden is probably much higher than current estimates suggest. This paper models the burden of MiP and associated foetal losses and maternal deaths, in rural Madhya Pradesh, India.

**Methods:**

Number of pregnancies per year was estimated from the number of births and an estimate of pregnancies that end in foetal loss. The prevalence of MiP, risk of foetal loss attributable to MiP and case fatality rate of MiP were obtained from the literature. The estimated total number of pregnancies was multiplied by the appropriate parameter to estimate the number of MiP cases, and foetal loss and maternal deaths attributable to MiP per year. A Monte Carlo simulation sensitivity analysis was done to assess plausibility of various estimates obtained from the literature. The burden of MiP in tribal women was explored by incorporating the variable prevalence of malaria in tribal and non-tribal populations and in forested and non-forested regions within Madhya Pradesh.

**Results:**

Estimates of MiP cases in rural Madhya Pradesh based on the model parameter values found in the literature ranged from 183,000–1.5 million per year, with 73,000–629,000 lost foetuses and 1,500–12,600 maternal deaths attributable to MiP. The Monte Carlo simulation gave a more plausible estimate of 220,000 MiP cases per year (inter-quartile range (IQR): 136,000–305,000), 95,800 lost foetuses (IQR: 56,800–147,600) and 1,000 maternal deaths (IQR: 650–1,600). Tribal women living in forested areas bore 30% of the burden of MiP in Madhya Pradesh, while constituting 18% of the population.

**Conclusion:**

Although the estimates are uncertain, they suggest MiP is a significant public health problem in rural Madhya Pradesh, affecting many thousands of women and that reducing the MiP burden should be a priority.

## Background

In high malaria-transmission settings such as much of Africa, the primary adverse outcomes of malaria in pregnancy (MiP) are low birth-weight babies and maternal anaemia. In low-transmission settings such as India, where the population is not sufficiently exposed to malaria to develop immunity by adulthood, MiP can have more serious consequences for both the foetus and mother including spontaneous abortion, stillbirth, and maternal death [1].

There is evidence of increasing malaria prevalence throughout India [2], which is thought to stem partially from economic development, which changes vector dynamics and human migration patterns, and partially from climate change, where recent models predict the spread of malaria into new regions [3]. Any spread of malaria in India will expose large populations, including pregnant women, to malaria [4]. A recent estimate suggests an annual incidence of malaria as high as 83 million cases per year [5]. However, there is a paucity of research into MiP in India and the lack of robust data on the burden has hampered progress in making evidence-based interventions to control MiP. This paper estimates the burden of MiP in Madhya Pradesh state in order to highlight the public health significance of MiP and to contribute to the debate on appropriate interventions to reduce the burden of MiP in India.

Madhya Pradesh was selected because it contributes the largest proportion of malaria cases among Indian states, roughly 27% [6], recent evidence suggests that malaria is on the rise there [7], and it has a large tribal population with a relatively high birth rate. Studies in Madhya Pradesh have found that between 80–87% of malaria cases were caused by *Plasmodium falciparum *[7], and most research on MiP has focused on this species. Given limited data on differences in maternal and foetal outcome of *P. falciparum *vs. *Plasmodium vivax *infection, for the purposes of this paper, the two are not distinguished between.

Rural women only are analyzed, as malaria incidence is negligible in urban areas of Madhya Pradesh. Therefore, the total population denominator only includes the rural population. Due to lack of separate data, this analysis of Madhya Pradesh includes the population of Chhattisgarh as well (Chhattisgarh became an independent state in 2000, however was previously part of Madhya Pradesh).

## Methods

The number of MiP cases per year was estimated by the following model:

Foetal loss rate (y) = proportion of pregnancies ending in spontaneous abortion + percent of pregnancies ending in stillbirth = 0.15+ 0.04 ≃ 0.20 Proportion of pregnancies ending in live births (b) = 1-"foetal loss rate" (y) = 1-0.2 = 0.8

Total pregnancies (TP) = Live Births (B)/b = B/0.8

Number of pregnant women with malaria (MiP) = TP* "prevalence of malaria in Pregnancy" (MiPr)

Foetal loss includes pregnancies lost to stillbirth and spontaneous abortion. A sensitivity analysis using a Monte Carlo simulation was done to assess the plausibility of the estimates of MiP cases obtained by this model. The Monte Carlo functions in the Berkeley Madonna Modeling Software [8] were used to test the effect of changing values of multiple parameters included in the model at once by randomly selecting from within a range of values of each variable every time the model was run to estimate the MiP cases, foetal loss and maternal deaths. The model was extended to incorporate the variation in the prevalence of malaria in tribal and non-tribal populations and in forested and non-forested regions within Madhya Pradesh.

The following input data for the model were estimated from the literature: (1) number of live births in Madhya Pradesh; (2) prevalence of MiP; (3) rates of stillbirth, spontaneous abortion and case fatality rate for pregnant women with malaria; (4) birth rates and prevalence of malaria in tribal women; (5) birth rates and prevalence of malaria in forest and non forest areas.

Using the 2001 census data, the number of births per year in Madhya Pradesh as a whole was estimated to be 2,287,000 [9,10]. There are limited data on foetal loss as it is very difficult to collect data on these outcomes due to their sensitive nature and problems with recall [11]. For the purposes of this paper, foetal loss includes spontaneous abortion (defined as foetus lost before 28 weeks) and stillbirths (defined as foetus lost between 28–36 weeks). A back-calculation to the total number of pregnancies, based on known number of births and the estimate of pregnancies lost to stillbirth and spontaneous abortion was made The reported national still birth rate in India is 39/1,000 [12]. The ratio of pregnancies to births in the general population was assumed to be roughly 1.25, to take into account an estimated global spontaneous abortion rate of 15% [13] and estimated Indian stillbirth rate of 4% [12]. To focus on rural populations alone, roughly 16 million people were subtracted from the total population of Madhya Pradesh, as this is the estimated urban population from the 2001 census [10]. All outcomes from modeling are presented rounded to three significant figures since these are estimates based on 500 trials and therefore are meant to show rough averages.

## Results

Applying the reported Annual Parasite Incidence (API) in 2006 in Madhya Pradesh, which is 1.28 cases of malaria per 1000 people per year [14], gives an estimated 2,600 cases of MiP per year. However the prevalence of MiP measured in health facility based studies in Madhya Pradesh was substantially higher, and varied remarkably ranging from 6.4% to 55% [15-19] (Table 1).

**Table 1:** Prevalence of malaria in pregnancy in Madhya Pradesh

**Year of study**	**Type of study**	**Definition of MiP**	**Prevalence of MiP**
2006 [15]	Cohort of women attending an antenatal clinic and/or delivering in a health facility	Presence of peripheral parasitaemia during pregnancy or at delivery	6.4%
2002–2004 [16]	Cohort of women delivering at two hospitals	Placental parasitaemia	11% (site 1) 14% (site 2)
1992–1995 [17]	Cohort of women attending ANC clinic	Peripheral parasitaemia	17%
1998 [18]	Cross sectional survey of pregnant tribal women in rural villages	Peripheral parasitaemia	39%
1997–1998 [19]	Cross sectional survey during an epidemic	Peripheral parasitaemia	55%

The rates of adverse maternal and foetal outcomes in women with malaria reported from India are summarized in Table 2. The study by Konar *et al *[20] is hospital based and thus the reported adverse outcomes of MiP are likely to be overestimates because women with mild malaria tend to be treated as outpatients. The proportion of pregnant women developing severe disease from malaria infection is speculated at around 5%, although the exact proportion of pregnant women with malaria infection becoming severe cases is unknown. Thus the rates of maternal and foetal adverse outcomes found in hospital based studies probably relate to 5% of the self-selected pregnant women with severe malaria infection admitted to hospitals [21]. This analysis assumes that the case fatality rate for pregnant women with malaria (8.4%) observed by Konar et al [20] relates to a 5% of pregnant women with severe malaria who use hospital services, therefore, the case fatality rate in pregnant women with malaria in general will be 0.84%, or 840 maternal deaths/100,000 pregnant women with malaria.

**Table 2:** Rates of adverse pregnancy outcomes attributable to MiP in India

**Year of study**	**Study population**	**Sample size**	**Adverse outcomes**		
			
			**Spontaneous Abortion**	**Still birth**	**Case Fatality Rate**
2001–2002 [20]	Pregnant women with peripheral parasitaemia at 9 health centers across India	215	32%	8%	8,400/100,000
2002–2004 [16]	Pregnant women with peripheral parasitaemia delivering at a hospital	209	3%	4%	NA
1997–1998 [19]	Pregnant women with peripheral parasitaemia in a tribal area during malaria epidemic	274	2%	2%	7,000/100,000

NA: not available

The study by Singh *et al *in 2002–2004 [16] was conducted in an urban teaching hospital and is therefore not generalizable to the rural populations. The study by Singh *et al *in 1997–98 [19] took place in a tribal population during a malaria epidemic and, therefore, estimates maternal and foetal outcomes attributable to malaria in extreme conditions.

**Figure 1:**
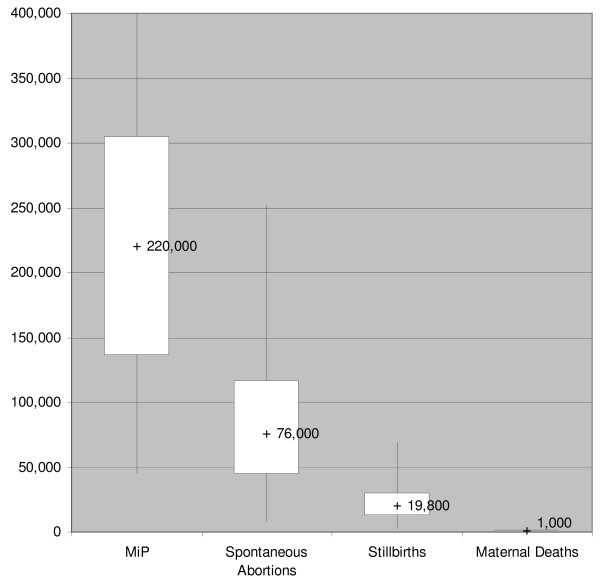
**Distribution of estimates of malaria in pregnancy cases, spontaneous abortions, still births and maternal deaths based on Monte Carlo Simulation: minimum, maximum, upper and lower quartiles, and median values of each outcome in 500 runs of the model**.

The estimates of cases of MiP in Madhya Pradesh obtained based on the model parameters values found in the literature, ranged from 183,000 to 1,572,000; foetal loss from 73,000 to 629,000 and maternal death from 1,500 to 12,600 (Table 3). The ranges of values of variables included in the Berkeley Madonna model to estimate the cases of MiP, foetal loss and maternal death are shown in Table 4. Even though estimates in the literature had a wider range, MiP prevalence was limited to between 2% and 14% as it is assumed that values above or below these values will be inconsistent with the current level of malaria transmission in Madhya Pradesh. Spontaneous abortion and stillbirth ranges were set 50% above and below the values reported by Konar *et al *[20]. For reasons discussed above, 10% of the Konar estimate for case fatality rate in pregnant women with malaria (8.4%) was used, and the range varied from half to twice that amount [20]. The ranges of MiP, births in MiP, maternal deaths and foetal loss due to MiP derived from running the model 500 times is shown in (Figure 1 and Table 5). Median annual values estimated were 220,000 for MiP cases, 19,800 stillbirths, 76,000 spontaneous abortions and 1,000 deaths in pregnant women with malaria.

**Table 3:** Estimates of annual cases of MiP, and fetal loss and maternal deaths attributable to MiP in Madhya Pradesh

**Study year & reference**	**Prevalence of MiP**	**MiP cases per year^1^**	**Fetal loss attributable to MiP^2^**	**Maternal Deaths attributable to MiP^3^**
Ahmed et al (2007) [15]	**6.4%**	183,000	73,000	1,500
Singh et al (2005) [16]	11–14% *	372,000	149,000	3,000
Singh et al (1999) [17]	**17%**	486,000	194,000	4,000
Korenramp et al (2004) [5]	**30%**	858,000	343,000	7,000
Singh et al (1998) [18]	**39%**	1,115,000	446,000	9,000
Singh et al (2001) [19]	**55%**	1,572,000	629,000	12,600

*MiP is defined as placental malaria

^1^MiP cases = (number of births * 1.25 *prevalence of MiP)

^2 ^Fetal loss attributable to MiP = (MiP cases * fetal loss rate attributable to MiP) [20]

^3^Maternal deaths associated with to MiP = (MiP cases * Case fatality rate of MiP) [20]

**Table 4:** Ranges of values of variables used in Berkeley Madonna Model*

**Variable**	**Lowest Value**	**Highest Value**
**MiP Prevalence**	0.02	0.14
**MiP Stillbirth rate**	0.02	0.08
**MiP Spontaneous abortion**	0.15	0.6
**MiP Case Fatality Rate**	0.042	0.168

* Ranges based on values found in the literature and applied to the Berkeley Madonna Model

**Table 5:** Output from Monte Carlo simulation using Berkeley Madonna Model*

	**MiP**	**Spontaneous Abortions**	**Stillbirths**	**Maternal Deaths**
**Median**	220,000	76,000	19,800	1,000
**Lower quartile**	136,000	44,600	12,200	650
**Minimum**	45,600	8,400	2,900	150
**Maximum**	506,000	252,000	69,000	3,500
**Upper Quartile**	305,000	117,000	30,600	1,600

*Berkeley Madonna Input

Total rural population (TPop) = 74481000–15967145

Crude birth rate (CBR) = random (0.0307, 0.04376)

Live Births (B) = TPop*CBR

y = proportion of pregnancies ending in spontaneous abortion (SA) + proportion of pregnancies ending in stillbirth (SB)

Total pregnancies (TP) = B/(1-y)

MiP = TP*PrevMiP

MiPSB = MiP*MiPSBr

MiPSA = MiP*MiPSAr

SevereMiP = 0.05*MiP

MiPMM = SevereMiP*MiPcase fatality rate

PrevMiP = random (0.02, 0.14)

MiPSAr = random (0.15, 0.6)

MiPSBr = random (.04, 0.16)

MiPMMr = random (0.042, 0.168)

SB = random (0.02, 0.08)

SA = random (0.075, 0.30)

Past research has suggested that tribal populations, forest cover, and malaria incidence are strongly correlated [2,22,23]. A study by Biswas [24] in a tribal population in Madhya Pradesh found a crude birth rate of 43/1000 and stillbirth rate of 270/1000 in tribal populations, compared a birth rate of 31/1000 and stillbirth rate of 39/1000 in the general population. Sharma *et al *[22] found an API of 284/1,000 population per year in highly forested and 32/1,000 in less forested regions, and that 40% of Madhya Pradesh is highly forested. Given that exact population distribution within forested regions is not known, this analysis assumes that all tribal people live in the forests, and thereby the differential impact of malaria on tribal populations can be calculated (Table 6). The tribal populations, which constitute only 18% of the total population, appear to shoulder as much as 30% of the MiP burden in Madhya Pradesh.

**Table 6:** The burden of MiP in tribal and non-tribal populations, Madhya Pradesh, 2006

	**Tribal Population**	**Non-tribal Population**	**Whole population of Madhya Pradesh**
**Annual parasite Prevalence (API) **[22]	284/1000	32/1000	158/1000
**Population in 2006**	13,721,000 **(18%)**	60,760,000 **(82%)**	74,481,000 **(100%)**
**Number of pregnancies**	721,000 **(26%)**	2,023,000 **(74%)**	2,744,000 **(100%)**
**Number MiP cases**	205,000 **(30%)**	88,000 **(70%)**	292,000 **(100%)**

## Discussion

The prevalence of MiP is poorly characterized, leading to a wide range of values in the literature and a consequent wide range in the estimates of MiP cases in Madhya Pradesh. As stillbirths, spontaneous abortions and maternal deaths are difficult to measure at a population level [25], the available data are unlikely to be accurate. The Berkeley Madonna model allows for these variations and performed a multifaceted sensitivity analysis. The estimates that occur the most frequently when the model was run many hundred times helped to narrow the width of potential ranges of the magnitude of each output. This model helped to elucidate the potential range of estimates incorporating all variables, but also the most likely level of burden given known estimates. The median values of 220,000 MiP cases (inter-quartile range (IQR): 136,000–305,000) and 95,800 lost foetuses (IQR: 56,800–147,600) are probably plausible estimates. Even with the fairly conservative estimates for case fatality rate attributable to MiP, the median estimate of 1,000 maternal deaths annually attributable to MiP (IQR: 650–1,600) is a huge burden in the state.

These rates are much higher than current official estimates suggest, although lower than estimates in the literature since these studies were conducted in hospital settings and therefore unrepresentative of the state as a whole. The maternal mortality ratio in Madhya Pradesh is estimated to be between 540–700/100,000 live births [25], which translates into a total of 12,350 maternal deaths (if the lower estimate of 540/100,000 is used). This would mean that roughly 8% of maternal deaths in Madhya Pradesh may be associated with malaria. As another example, if the state-wide stillbirth rate is 39/1,000 births, then there would be an extrapolated 93,000 stillbirths annually in Madhya Pradesh as a whole. This model estimates a median number of 19,800 stillbirths due to MiP, which would suggest roughly 21% of stillbirths in this state are due to MiP. Both of these extrapolations suggest that large proportions of state-wise adverse pregnancy outcomes are associated with malaria. Given that both stillbirth and maternal mortality rates are difficult to measure in the general population, it is possible that the denominator state-wide number of stillbirths and maternal deaths is an underestimate. Therefore, the proportion due to MiP could actually be lower than the above percentages suggest. Regardless of the underlying denominator of adverse outcomes in the general population, this model suggests that malaria is a significant factor in foetal and maternal deaths in this region. It is also important to keep in mind that these results suggest correlation between MiP and adverse pregnancy outcomes, not necessarily causality.

This analysis also highlights the importance of considering the heterogeneity of the burden of MiP within Madhya Pradesh. MiP in tribal women is disproportionately high due to higher birth rates and a greater malaria prevalence linked to forest residence. A large proportion of India's tribal populations live in central/central-eastern India [26] and a recent study found strong correlation between tribal populations, malaria incidence (especially *P. falciparum*) and forest cover [23]. Therefore, it is essential to look in depth at the tribal populations and local environmental conditions to understand fully the burden MiP and the means of controlling it in this population. Given that Madhya Pradesh has received attention due to its high foetal and maternal death rates, focusing on preventing and treating malaria might have significant benefits.

Control strategies for MiP focus primarily on Africa because the evidence base for the interventions is from Africa. There is no reported evidence on the efficacy, effectiveness or cost effectiveness of control measures of MiP in India. Currently in India, chemoprophylaxis with chloroquine is recommended for pregnant women living in high risk areas with low drug resistance, and chloroquine plus proguanil in highly resistant areas [27]. Recently intermittent preventive treatment for pregnant women in high risk using two doses of sulphadoxine-pyrimethamine was recommended by the Indian Drug Policy Working Group [2]. A review of data on efficacy of anti-malarials throughout India found that the majority of studies reported more than a 25% failure rate for chloroquine against *P. falciparum *by day 28 post treatment [2]. Research in Madhya Pradesh suggested that 23% of malaria cases were resistant to chloroquine [7]. Given that there is a huge burden of MiP in Madhya Pradesh in general, and in the tribal population in particular, research into appropriate interventions to reduce the burden of MiP needs to be conducted.

This paper has not addressed or modeled the impact of MiP on LBW, preterm delivery, and maternal anaemia in this population. Past research has suggested that in high transmission settings, malaria as much as doubles the risk of LBW and is responsible for an estimated 26% of maternal anaemia [28]. High and low transmission settings have been found to have comparable LBW and maternal anaemia rates, although, as this paper highlights, low-transmission settings have the added burdens of maternal death and foetal loss [29]. Therefore, although not explicitly modeled here, it can be posited that LWB and maternal anaemia rates would be similar in Madhya Pradesh as in other malarious areas, and is an added cause of concern. Not only is MiP a pressing health concern today, but the importance of MiP will only continue to grow in India due to the combined threat of population growth and climate change [4]. More research is needed to pinpoint the number of women at risk, the most vulnerable among these women, and how to better protect them with appropriate treatment and prevention both today and in the future.

The data presented in this paper, though not providing precise estimates of MiP in Madhya Pradesh, show the potential magnitude of cases of MiP, as well as malaria related foetal and maternal loss. This is the first step towards sensitizing the research community and advocating that sponsors of research build the evidence base on the burden of MiP in India so as to better evaluate potential interventions.

## Competing interests

The authors declare that they have no competing interests.

## Authors' contributions

ND conducted the background review, did the mathematical modeling, and wrote the manuscript. DC provided guidance in concept and design of research. DC also read, thoroughly edited and provided experienced advice and support with every draft.

OC reviewed manuscript in depth with primary authors and advised on some parameter calculations. NS reviewed manuscript and provided valuable comments. DG reviewed manuscript and provided valuable comments. KT reviewed manuscript and provided valuable comments. AD reviewed manuscript and provided valuable comments
